# Determinants of point-of-care ultrasound lung sliding amplitude in mechanically ventilated patients

**DOI:** 10.1186/s13089-023-00326-5

**Published:** 2023-05-23

**Authors:** David N. Briganti, Christine E. Choi, Julien Nguyen, Charles W. Lanks

**Affiliations:** 1 Department of Medicine, Harbor-UCLA Medical Center, Torrance, CA USA; 2 Division of Pulmonary and Critical Care Physiology and Medicine, Harbor-UCLA Medical Center, 1000 W. Carson Street, Box 405, Torrance, CA 90509 USA; 3 Lundquist Institute for Biomedical Innovation, Torrance, CA USA

**Keywords:** Ultrasound, POCUS, Point-of-care ultrasound, Lung sliding, Lung ultrasound, Lung sliding amplitude, Quantification, Mechanical ventilation

## Abstract

**Background:**

Although lung sliding seen by point-of-care ultrasound (POCUS) is known to be affected to varying degrees by different physiologic and pathologic processes, it is typically only reported qualitatively in the critical care setting. Lung sliding amplitude quantitatively expresses the amount of pleural movement seen by POCUS but its determinants in mechanically ventilated patients are largely unknown.

**Methods:**

This was a single-center, prospective, observational pilot study examining 40 hemithoraces in 20 adult patients receiving mechanical ventilation. Each subject had lung sliding amplitude measured in both B-mode and by pulsed wave Doppler at their bilateral lung apices and bases. Differences in lung sliding amplitude were correlated with anatomical location (apex vs base) as well as physiologic parameters including positive end expiratory pressure (PEEP), driving pressure, tidal volume and the ratio of arterial partial pressure of oxygen (PaO_2_) to fraction of inspired oxygen (FiO_2_).

**Results:**

POCUS lung sliding amplitude was significantly lower at the lung apex compared to the lung base in both B-mode (3.6 ± 2.0 mm vs 8.6 ± 4.3 mm; *p* < 0.001) and the pulsed wave Doppler mode (10.3 ± 4.6 cm/s vs 13.9 ± 5.5 cm/s; *p* < 0.001) corresponding to expected distribution of ventilation to the lung bases. Inter-rater reliability of B-mode measurements was excellent (ICC = 0.91) and distance traversed in B-mode had a significant positive correlation with pleural line velocity (*r*^2^ = 0.32; *p* < 0.001). There was a non-statistically significant trend towards lower lung sliding amplitude for PEEP ≥ 10 cmH_2_O, as well as for driving pressure ≥ 15 cmH_2_O in both ultrasound modes.

**Conclusion:**

POCUS lung sliding amplitude was significantly lower at the lung apex than the lung base in mechanically ventilated patients. This was true when using both B-mode and pulsed wave Doppler. Lung sliding amplitude did not correlate with PEEP, driving pressure, tidal volume or PaO_2_:FiO_2_ ratio. Our findings suggest that lung sliding amplitude can be quantified in mechanically ventilated patients in a physiologically predictable way and with high inter-rater reliability. A better understanding of POCUS derived lung sliding amplitude and its determinants may aid in the more accurate diagnosis of lung pathologies, including pneumothorax, and could serve as a means of further reducing radiation exposure and improving outcomes in critically ill patients.

**Supplementary Information:**

The online version contains supplementary material available at 10.1186/s13089-023-00326-5.

## Introduction

Point-of-care ultrasonography (POCUS) refers to the acquisition and real-time interpretation of ultrasound imaging data at the patient’s bedside. It is essential to the care of critically ill patients and may help narrow the differential diagnosis of shock and respiratory failure. Daniel Lichtenstein coined the term “lung sliding” to describe the ultrasound appearance of the parietal pleura (lining the inner chest wall) and visceral pleura (lining the surface of the lung) moving against one another during ventilation (Additional file 1: Video S1) [1–3]. The lung sliding sign is abolished by pneumothorax [1] due to the near complete reflection of ultrasound waves by free pleural air. However, absent lung sliding may also be seen in other processes affecting lung compliance and regional ventilation including acute respiratory distress syndrome (ARDS), pulmonary fibrosis, chronic obstructive pulmonary disease (COPD) and mechanical ventilation with high levels of positive end expiratory pressure (PEEP) [4–7]. Absent lung sliding has also been noted at the lung apex relative to the base in mechanically ventilated patients [8].

As in the aforementioned studies, POCUS lung sliding is typically reported qualitatively as being absent or present, but it can be quantified with greater precision. Lung sliding amplitude expresses the amount of pleural movement seen by ultrasound and has been described both in terms of pleural distance travelled in B-mode (mm) [9] and maximum pleural velocity measured by Doppler (cm/s) [10]. However, it has never simultaneously been reported using both B-mode and pulsed wave Doppler in the same study and measurements by these two techniques have never been correlated.

The aim of this pilot study was to identify the determinants affecting POCUS lung sliding amplitude in mechanically ventilated patients. We hypothesized that the lung bases would have increased lung sliding amplitude compared to the lung apices due to preferential distribution of ventilation there and that measurements in B-mode would have a significant positive correlation with the lung sliding velocity measured by pulsed wave Doppler.

## Methods

### Study design and participants

This was a single center, prospective observational pilot study. The study population consisted of 40 hemithoraces in 20 adult patients admitted to the medical intensive care unit (ICU). Patients were eligible for inclusion if they were receiving mechanical ventilation and > 18 years of age. Patients at the end-of-life or with pathologies precluding accurate assessment of ultrasound lung sliding were excluded. The study protocol was approved by the Lundquist Institute for Biomedical Innovation’s Institutional Review Board and written informed consent was obtained from each subject or their legally authorized representative. Demographic information and physiologic parameters including PEEP, driving pressure, tidal volume and ratio of arterial partial pressure of oxygen to fraction of inspired oxygen (PaO_2_:FiO_2_ ratio) were collected.

### Point-of-care ultrasound protocol

Pleural ultrasonography (Sonosite X-porte; Fujifilm) was performed using a high frequency linear probe (L38xp; 15-6 MHz). Images were acquired at the lung apex at the second intercostal space in the anterior midclavicular line and the lung base at the lateral midaxillary line, one intercostal space above the hemidiaphragm. The transducer was oriented in the longitudinal plane with the indicator in a cephalad position [2]. Image depth was adjusted individually according to the degree of subcutaneous tissue superficial to the pleural space such that the pleural line was always positioned mid-screen.

**Fig. 1:**
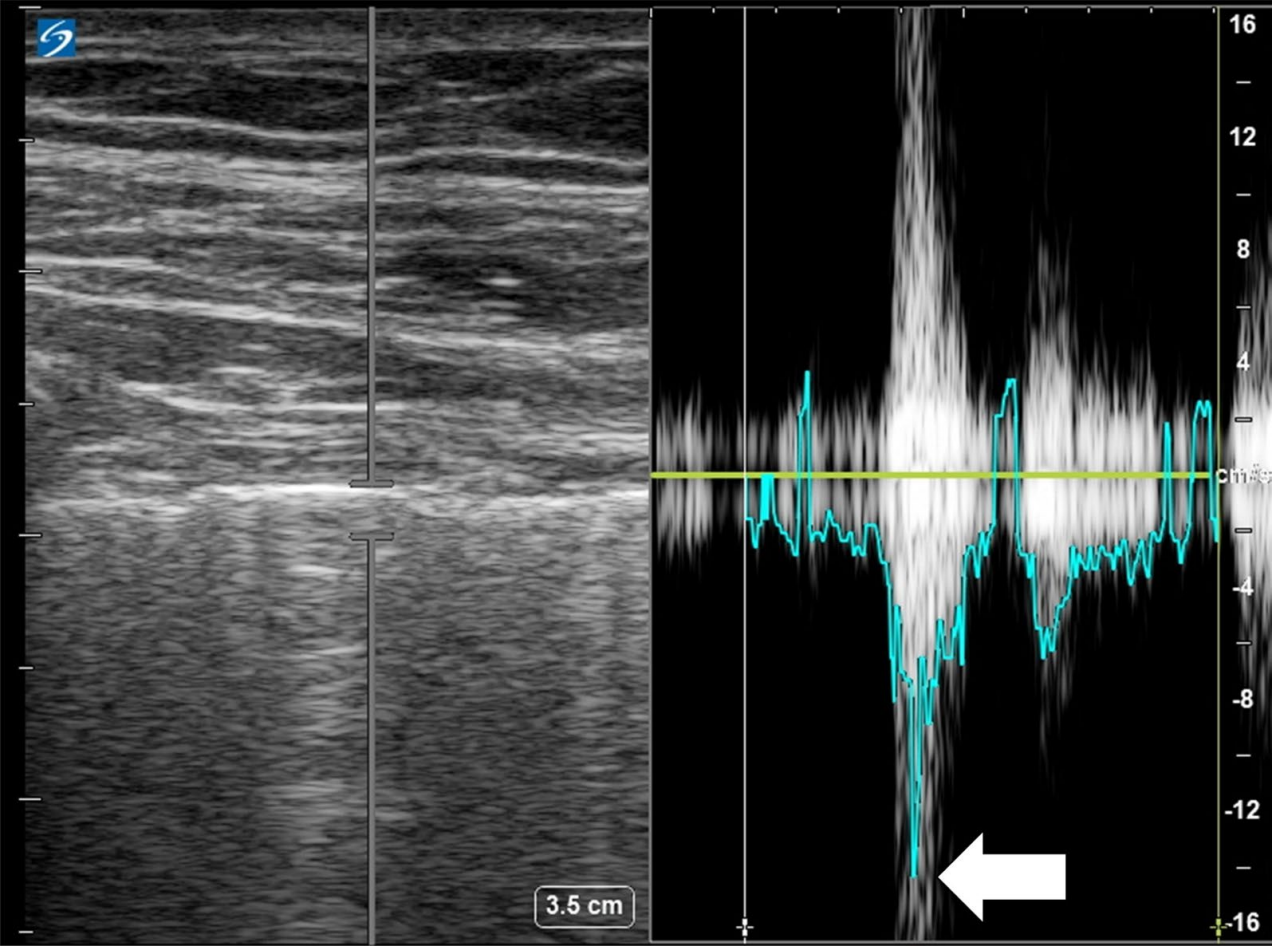
Example measurement of pleural velocity by pulsed wave Doppler. A maximum pleural velocity of 14.5 cm/s (white arrow) was measured with the Doppler reading gate positioned at the pleural line

In B-mode, a comet-tail artifact, also known as a B-line, or other visible pleural defect was identified by the operator and its maximum displacement over a single breath was measured in millimeters. To replicate real world POCUS conditions, displacement was measured manually without the aid of image analysis software (see Additional file 1: Video S1) and recorded in millimeters. Measurements were performed by two ultrasonographers (one expert and one novice) trained in the use of pleural ultrasound. It should be noted that while pleural defects tend to be easily visible in critically ill patients, it is likely that they occur less frequently in healthy lungs. Pulsed wave Doppler was used to automatically calculate maximum pleural line velocity using a Doppler reading gate of 2 mm in the same ultrasound view as was used for B-mode measurements (Fig. 1). As movement of the pleural line occurs at an angle of 90° to the ultrasound probe, a standardized angle correction of + 60° was used for pleural velocity measurements.

### Statistical analysis

Continuous variables were expressed as means with standard deviations. Differences between groups were assessed using Student’s *t*-test and Fisher’s exact test. Lung sliding amplitude at the lung apex and lung base was compared using both paired and unpaired *t*-tests. All reported *p* values are two-sided. Pearson correlation coefficients were used to express relationships between lung sliding amplitude, pleural line velocity and other recorded physiologic parameters. Inter-rater reliability in B-mode was assessed using intra-class correlation coefficients (ICC).

## Results

**Fig. 2:**
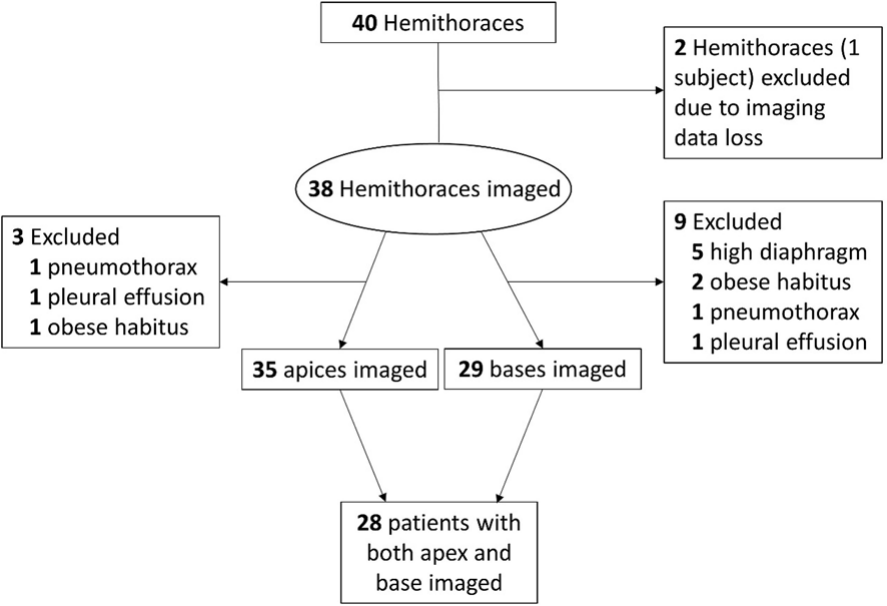
Reasons for excluded measurements at the lung apex vs base

The median age of patients studied was 56.5 years (interquartile range, 37 to 63) and 60% were male (Table 1). Indications for mechanical ventilation are shown in Table 1. Measurements could not be obtained in some patients due to superiorly displaced hemidiaphragm (*n* = 5), obese body habitus (*n* = 2), pneumothorax (*n* = 1) and large pleural effusion (*n* = 1). Lung sliding amplitude was successfully measured at the lung apex in 35 patients and at the lung base in 29 patients. Imaging at both the apex and base was obtained in 28 patients (Fig. 2).

**Table 1:** Patient characteristics

Age—year	54.6 ± 18.6
Male sex—%	60%
Indication for mechanical ventilation	
COVID-19 associated ARDS^†^—no	8
Cardiac arrest—no	3
Acute encephalopathy—no	3
Community acquired pneumonia—no	2
Heart failure exacerbation—no	1
Upper gastrointestinal bleeding—no	1
Acute liver failure—no	1
Neuromuscular weakness—no	1
Characteristics of mechanical ventilation	
Tidal volume—mL	412 ± 50
PEEP^‡^—cmH_2_O	7 ± 4
Driving pressure—cmH_2_O	18 ± 10
PaO_2_:FiO_2_^§^	240 ± 143

Plus–minus values are means ± SD

^**†**^Acute respiratory distress syndrome

^**‡**^Positive end expiratory pressure

^§^Ratio of arterial partial pressure of oxygen to fraction of inspired oxygen

**Fig. 3:**
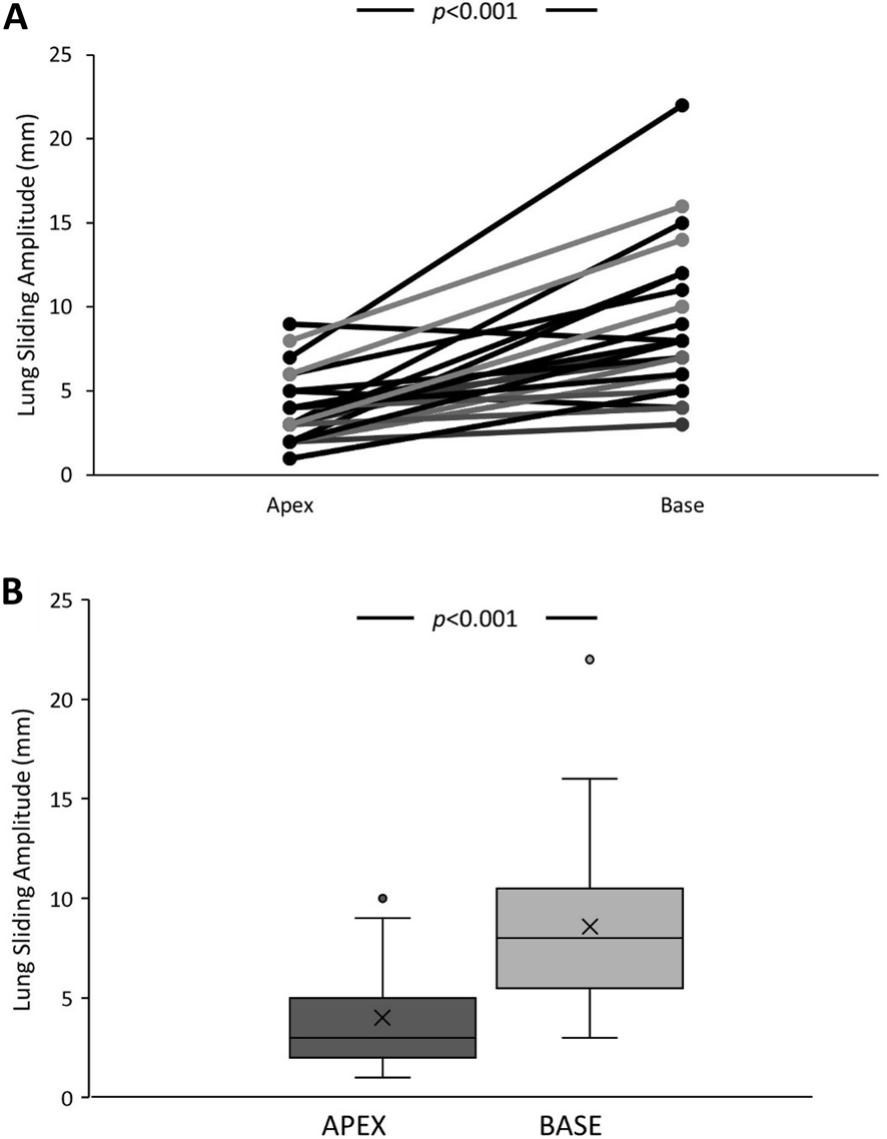
Difference in B-mode lung sliding amplitude (mm) between the lung apex and lung base expressed using **A** paired and **B** unpaired *t*-tests

POCUS lung sliding amplitude measured in B-mode was significantly lower at the lung apex compared to the lung base (3.6 ± 2.0 mm vs 8.6 ± 4.3 mm; *n* = 28; *p* < 0.001) (Fig. 3). According to categories previously described by Lichtenstein, lung sliding amplitude was further categorized as “normal” if > 10 mm, “significantly reduced” between 4 and 10 mm, “quite abolished” between 1 and 3 mm and “fully abolished” if there was no visible pleural movement (0 mm) [9]. Lung sliding amplitude was more likely to be “quite abolished” at the lung apex (54% apex vs. 3% base; *p* < 0.001) and less likely to be “normal” (0% apex vs 24% base; *p* < 0.001). Excluding one patient with undiagnosed pneumothorax, no patients had “fully abolished” lung sliding at any location (Table 2). For B-mode measurements, inter-rater reliability was excellent with an intra-class correlation coefficient (ICC) of 0.91.

**Table 2:** Lung sliding amplitude study results

Ultrasound mode	Apex	Base	*p*-value
B-mode—mm	3.6 ± 2.0	8.6 ± 4.3	< 0.001
Pulsed wave Doppler—cm/s	10.3 ± 4.6	13.9 ± 5.5	< 0.001
Reduction in lung sliding amplitude (B-mode)			
Normal—no. (%)	0/34	0/20	n/a
Significant reduction^†^—no./total (%)	16/34 (47)	19/20 (97)	< 0.001
Quite abolished^‡^—no./total (%)	18/34 (53)	1/20 (3)	< 0.001
Abolished^§^—no./total (%)	0/34	0/20	n/a

Plus–minus values are means ± SD

^**†**^4–10 mm; ^**‡**^1–3 mm; ^§^0 mm

**Fig. 4:**
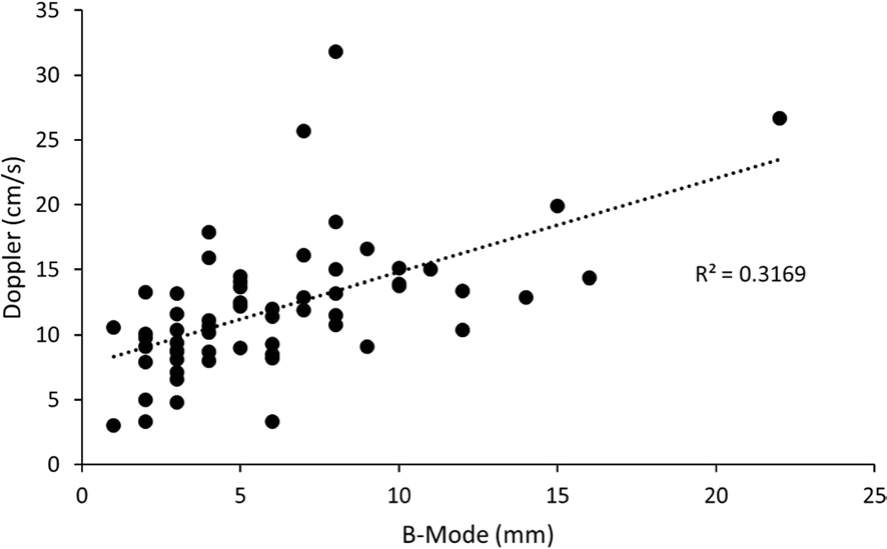
Scatterplot and superimposed best fit line (dashed) showing correlation between lung sliding amplitude measured in B-mode and pleural line velocity measured in the pulsed wave Doppler mode (*r*^2^ = 0.32, *p* < 0.001)

Pleural line velocity measured in the pulsed wave Doppler mode was also significantly lower at the apex compared to the base (10.3 ± 4.6 cm/s vs 13.9 ± 5.5 cm/s; *n* = 27; *p* < 0.001). Distance traversed in B-mode had a statistically significant positive correlation with pleural line velocity (*r*^2^ = 0.32; *p* < 0.001) (Fig. 4).

**Fig. 5:**
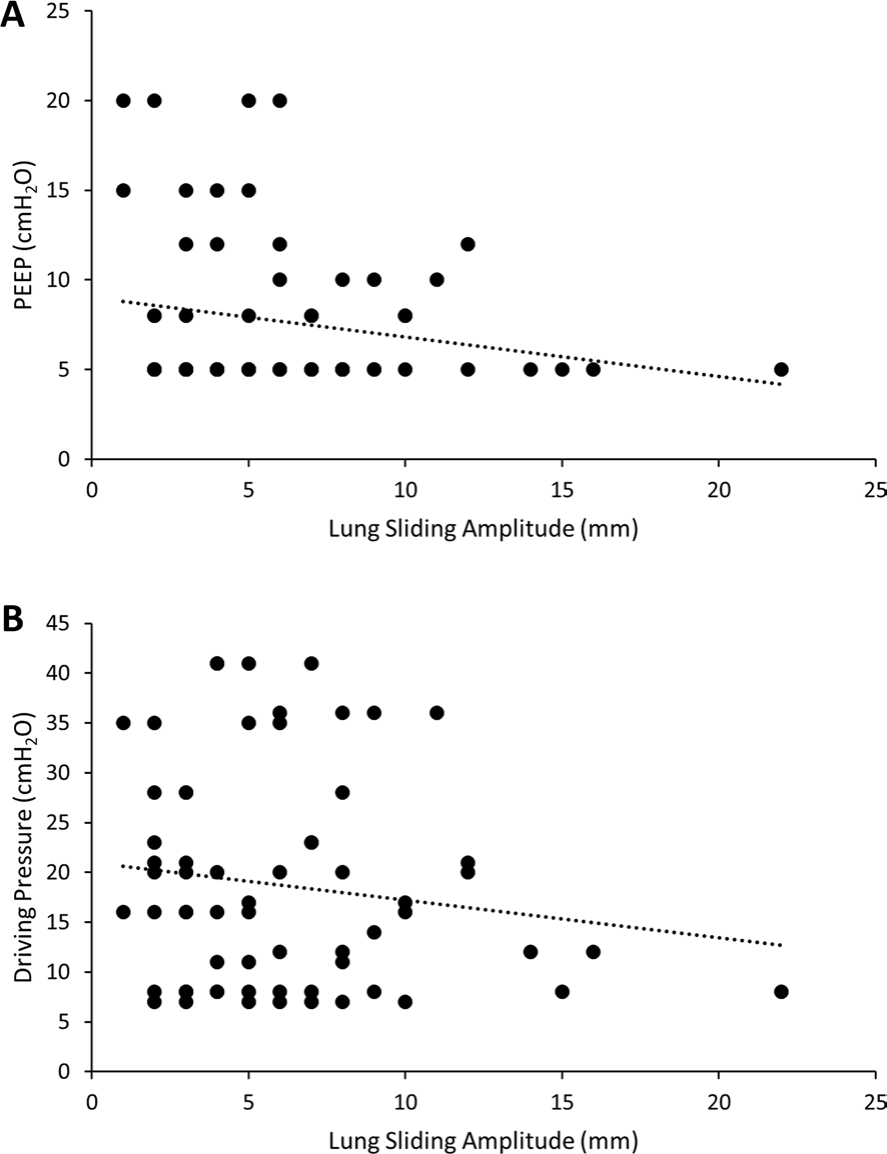
Scatterplot and superimposed best fit line (dashed) showing correlation between B-mode lung sliding amplitude (mm) and **A** positive end expiratory pressure (cmH_2_O) and **B** driving pressure (cmH_2_O)

There was a non-statistically significant trend towards lower lung sliding amplitude for PEEP ≥ 10 cmH_2_O compared with PEEP < 10 cmH_2_O in both B-mode (5.4 ± 3.3 mm vs 6.3 ± 4.2 mm; *p* = 0.42) and the pulsed wave Doppler mode (10.1 ± 4.6 cm/s vs 12.4 ± 5.4 cm/s; *p* = 0.14) (Fig. 5A). There was also a trend toward lower lung sliding amplitude for driving pressure ≥ 15 cmH_2_O compared with driving pressure < 15 cmH_2_O in both B-mode (5.3 ± 3.1 mm vs 7.2 ± 4.8 mm; *p* = 0.06) and the pulsed wave Doppler mode (11.3 ± 5.4 cm/s vs 12.5 ± 5.1 cm/s; *p* = 0.36) (Fig. 5B). There were no correlations with tidal volume or PaO_2_:FiO_2_ ratio.

## Discussion

POCUS lung sliding amplitude was significantly greater at the lung base compared to the lung apex in mechanically ventilated patients but did not have a statistically significant correlation with other physiologic parameters including PEEP, driving pressure, tidal volume and PaO_2_:FiO_2_ ratio. Ventilation is known to be approximately 50% lower at the lung apex compared to the base in normal lungs [11, 12]. Due to the effects of gravity, the cumulative weight of lung parenchyma at the lung base is greater than at the apex, leading to increased pleural pressure and lower resting lung volume. During inspiration, these lower volume, more compliant alveoli experience a greater change in volume compared to those at the apex and, thus, increased ventilation [13, 14]. By studying patients receiving mechanical ventilation with set tidal volumes and semi-upright positions, we were able to utilize expected physiologic differences in regional ventilation between the lung apex and base to demonstrate that subtle differences in lung sliding amplitude can be detected and quantified by pleural ultrasound.

Reduced apical lung sliding amplitude was seen in both B-mode and pulsed wave Doppler. Previous studies examining lung sliding amplitude in mechanically ventilated patients were done using tissue Doppler imaging (TDI) [10] or pulsed wave Doppler [15]. By utilizing B-mode, our study best reflects standard use of lung ultrasound in critically ill patients, such as described by the BLUE-protocol (Bilateral Lung Ultrasound in Emergency) [2]. This remarkably simple measurement has been described before by Lichtenstein [9], but perceived problems with reproducibility and lack of clinical application have limited its widespread use. As expected, B-mode and pulsed wave Doppler measurements had a statistically significant positive correlation. That correlation may be further strengthened in future studies by analyzing the velocity–time integral of the Doppler tracing (i.e., distance traversed = ∫*v* d*t*) yielding a Doppler derived measurement of distance that might be more comparable to those obtained in B-mode. Although pulsed wave Doppler measurement has the potential to be less subjective than manual measurement of pleural movement in B-mode, B-mode measurements in our study had excellent inter-rater reliability.

Knowledge of baseline differences in POCUS lung sliding amplitude in critically ill patients will affect the specificity of the abolished lung sliding sign for detecting pneumothorax. For example, in patients with already reduced baseline lung sliding amplitude, false positivity rates for pneumothorax may be increased. Likewise, confirmatory imaging of the lung base in patients with absent apical lung sliding may be a physiologically sound method for confirming the diagnosis of pneumothorax in patients known to already have reduced apical ventilation.

According to prior studies, lung sliding amplitude is reduced by impaired ventilation [4–8]. In our study, this conclusion is further supported by lower lung sliding amplitude at the lung apex relative to the base. Pulmonary pathologies affecting ventilation, such as bacterial pneumonia, tuberculosis or a history of prior pleurodesis are also likely to affect regional lung sliding amplitude. Interestingly, vertical displacement of the pleural line has been shown to correlate with airflow limitation in patients with bronchospasm and could serve to complement horizontal lung sliding amplitude in patients with obstructive lung disease [16]. In light of our growing understanding of its applications, POCUS guided assessment of regional ventilation may represent an untapped well of information in critically ill patients. Further exploring analysis of pleural movement may aid in the ultrasound guided diagnosis of lung pathologies such as lobar pneumonia, COPD and ARDS. It may also help evaluate, or even predict, the effectiveness of therapies aimed at improving regional ventilation, such as prone positioning in ARDS.

We hypothesized that POCUS lung sliding amplitude would correlate with several important factors associated with patient outcomes in the ICU including PEEP, driving pressure, tidal volume and PaO_2_:FiO_2_ ratio. In patients with ARDS, increasing PEEP aids alveolar recruitment and improves the overall compliance of the respiratory system but could also cause regional overdistention [17]. Driving pressure (defined as the difference between alveolar pressure at the end of inspiration and PEEP) reflects reduced lung compliance and has been linked to increased mortality in ARDS [18]. In our study, there were trends towards decreased lung sliding amplitude at higher PEEP and driving pressure, but they were not statistically significant. In future studies, a larger population size may better describe this association.

We postulated that lung sliding amplitude would positively correlate with tidal volume, but this was not seen in our study. This was likely because of the lung protective ventilation strategies employed in our ICU with tidal volumes almost always set between 6 and 8 mL/kg of ideal body weight [18]. Because tidal volumes were standardized to predicted lung size for each patient, the distribution of values was small and differences in lung sliding amplitude relative to tidal volume were difficult to detect. Future studies should aim to capture greater differences in lung sliding amplitude at different tidal volumes within the same patient.

Lung sliding amplitude has been evaluated using tissue Doppler [10] and speckle tracking, particularly for the diagnosis of pneumothorax rather than assessment of regional ventilation [19–21]. Speckle tracking is a technique routinely used in echocardiography whereby tissue deformation is assessed by analysis of speckle pattern movement in a two-dimensional plane. Measurements by this modality are likely to correlate with the techniques employed in our study, but this hypothesis requires further investigation. Although speckle tracking is an intriguing means of precisely quantifying tissue movement, it requires specialized software not typically available to the POCUS practitioner.

This pilot study had several limitations. First, population size was small. Therefore, our results should be interpreted with caution and treated primarily as hypothesis generating. In the future, a larger number of subjects will be needed to corroborate our findings and better describe correlations between lung sliding amplitude, PEEP and driving pressure. Second, small sample size also affected our ability to study the effect of specific pulmonary pathologies on lung sliding amplitude. Third, because manual measurements of lung sliding amplitude were chosen over software based image analysis to replicate real world POCUS settings, there may have been some subjectivity in image interpretation. However, this limitation was mostly addressed by calculating inter-rater reliability. Also related to ultrasound technique, although automatic angle correction used was used in our Doppler assessments, we are still likely to have underestimated the true velocity of pleural movement occurring perpendicular to the ultrasound probe. Fourth, differences in regional ventilation in this study were assumed based on patient positioning. Future studies correlating lung sliding amplitude to known methods of ventilation quantification will be required. Fifth, the anatomic windows in which ultrasound images were captured were based arbitrarily on previously suggested protocols [2]. In future studies, the locations in which minimal and maximal lung sliding amplitude is observed may provide more accurate insight into the true amount of regional ventilation there. Finally, because our study focused only on patients receiving mechanical ventilation, extrapolation to non-intubated patients is limited.

## Conclusion

In this pilot study, POCUS lung sliding amplitude was significantly lower at the lung apex compared to the lung base in mechanically ventilated patients but did not correlate with PEEP, driving pressure, tidal volume or PaO_2_:FiO_2_ ratio. This was true when using both B-mode and the pulsed wave Doppler mode. Our findings suggest that lung sliding amplitude can be quantified in a meaningful and physiologically predictable way in mechanically ventilated patients. A better understanding of lung sliding amplitude and its determinants may aid in the more accurate diagnosis of lung pathologies, including pneumothorax, and could serve as a means of further reducing radiation exposure and improving outcomes in critically ill patients.

## Supplementary Information

**Additional file 1: Video S1.** Example measurement of lung sliding amplitude in B-mode. A B-line is tracked over the course of a mechanical breathwith horizontal distance traversed in this example measured to be approximately 7 mm.

## Data Availability

The datasets used and/or analyzed during the current study are available from the corresponding author on reasonable request.

## Abbreviations

ARDS: Acute respiratory distress syndrome
BLUE: Bilateral lung ultrasound in emergency
COPD: Chronic obstructive pulmonary disease
FiO_2_: Fraction of inspired oxygen
ICC: Intra-class correlation coefficient
ICU: Intensive care unit
PaO_2_: Arterial partial pressure of oxygen
PEEP: Positive end expiratory pressure
POCUS: Point of care ultrasound
TDI: Tissue Doppler imaging
